# Novel R-CNN and transformer models for pollution impacts and land cover changes around iconic heritage sites in developing countries: a case study

**DOI:** 10.1038/s41598-025-27155-x

**Published:** 2025-12-03

**Authors:** Satyajit Ghosh, B. Ashok, L. Agilandeeswari, M. Prabukumar, Ariful Rahaman, Abeer Mathur, Abhinav Sudhakar Dubey, Eishani Purohit, Visakh Gangadharan

**Affiliations:** 1 VIT Vellore, Vellore, India; 2 Leeds University, Leeds, UK

**Keywords:** Computer vision, Particle drop size distribution, Transformer, Vehicular pollution, Heritage conservation, Sustainability, Climate sciences, Engineering, Environmental sciences, Environmental social sciences

## Abstract

Irreversible Land Use Land Cover changes around iconic heritage sites in south Asian countries, precipitated by an ever-increasing urban density build up, is a serious cause of concern. Some of the monuments are UNESCO world heritage sites and are currently under threat. Heavy vehicular traffic and unregulated encroachments over several south Asian cities, including Indian cities have led to particulate pollution build up around iconic heritage structures. This study shows that emissions from diesel engines operating around a network of roads around the 2000-year-old Meenakshi Amman Temple complex, emit soot and black carbon particles in the accumulation mode (0.2–0.5 μm) which remain airborne for over a week and eventually soil and mar the beauty of the temple’s iconic tower gates (Gopurams). Over this size range the scavenging efficiency from natural rain showers is ~ 20%. Efficient scavenging depends not only on the rain drop size distribution, but also on the collected aerosol particle size distribution. The region of Madurai remains largely dry for 9 months in a year and municipal authorities must look for the use of pressure calibrated sprinklers optimised with the drop-size distribution of the airborne particles. Quantifying particle size distribution requires the use of in situ scanning mobility particle sizer spectrometers-SMPS -which is prohibitively expensive for developing nations. Creating artificial rain showers is an even more expensive process and is resorted to only in dire situations. From an analysis of European data repositories and with the use of the state-of-the-art Weather Research and Forecasting (WRF) model we show (i) a progressive loss of vegetative cover (and hence a decrease in the leaf area index, LAI) (ii) decrease in precipitation (iii) decrease in soil moisture and (iv) an increase in fractions of non-precipitating clouds over the region over a time period of 1940–2050. It is therefore clear that any economically affordable engineered solution must involve smart roadside sprinklers yielding a fine spray mist which will be able to capture these particles through efficient collision and coalescence until non-polluting vehicles are used. It is shown that when vehicles are in an idling mode (as is very often the case) the median size is at its broadest and progressively decreases with increasing vehicular operating loads. This study proposes a quick AI mediated method to specify the range of Sauter Mean Radii (SMR) for varying vehicular operations, completely circumventing the use of SMPS and geoengineered artificial rain showers. The drop size distribution (DSD) range will help in the choice of sprinkler attributes—a sprinkler yielding an assortment of millimetre sized droplets should scavenge much of the soot particles with moderate spray durations of the order of a few hours. Mask Recurrent-Convolutional Neural Networks(R-CNN) modelling was used to ascertain particle SMR. Future predictions spanning the years 2020–2050 using the Stacked Transformer model show a progressive decrease in the LAI and soil moisture and an aggravated increase in heat stress during the Representation Concentration Pathway (RCP) 8.5 high emissions scenario. Simulations are contrasted for business-as-usual scenarios (using polluting vehicles), with scenarios using a phased-out transition to electric vehicles (EVs) and it was ascertained that if municipal agencies replace diesel vehicles with EVs, then the above indicators significantly improved. These quick protocols can be used by government officials and other stakeholders to provide smart forecasts for Impact analyses (EIA) and cost savings assessments for calculating paybacks resulting from a partial transition to cleaner vehicular transport in the interim period. Estimates suggest benefits to the tune of millions of USD over a 5-year period vis-à-vis facade restoration ravaged by soot deposition.

**Supplementary Information:**

The online version contains supplementary material available at 10.1038/s41598-025-27155-x.

## Introduction

**Fig. 1:**
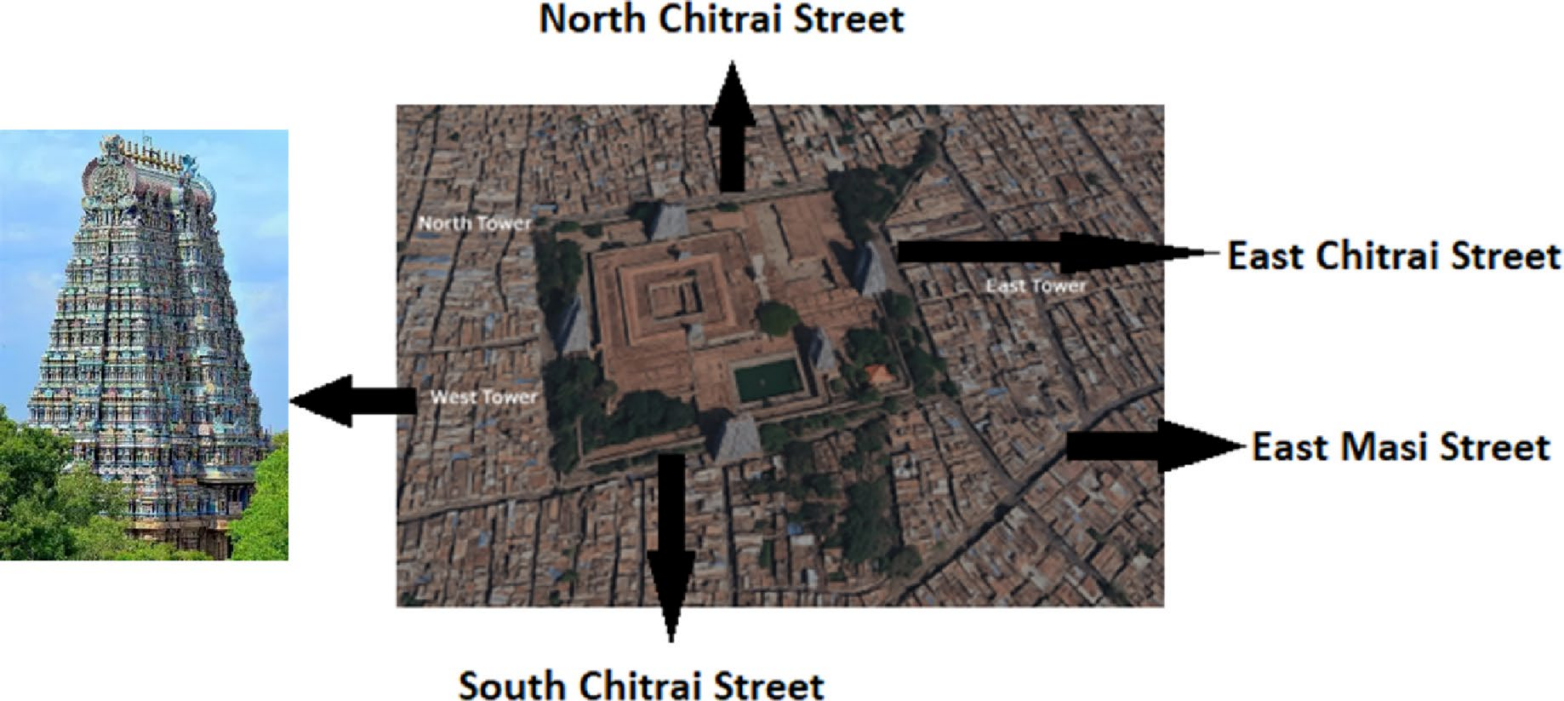
shows aTemple Tower and an aerial view of the complex. One can see the heavily built-up areas along with the busiest streets.

All of South Asia holds repositories of outstanding architectural heritage. India prides itself in its 43 UNESCO world heritage monuments and the state of Tamil Nadu alone has 4. The Tamil language is among the oldest languages in the world and stands shoulder to shoulder with Hebrew, Latin, Greek and Sanskrit. The world-renowned author William Dalrymple describes the richness of the Tamil Architectural heritage in glowing terms. There are only a handful of places in the world where ‘landscape and divinity are more closely linked than in south India’^1^. In Tamil Nadu’s sacred topography of the Deep South, every village hosts temple complexes. These structures and their precincts are regrettably not protected from the scourge of climate change induced weathering and erosion effects. Planned strategies are only just beginning to be put in place but are certainly not at a level comparable to conservation initiatives in the European Union. Currently, museum curators in India prioritize conservation of precious artefacts based on principles of material conservation. Temple facades however are exposed to the elements. The iconic Meenakshi Temple in Madurai is one such example and forms the piece d’ resistance of this study. The temple is embellished with intricately carved Gopurams (gateway towers), sculptures and frescoes that are over thousands of years old and spanning around 15 acres within the city of Madurai, is one of the largest temple complexes in India, attracting millions of pilgrims and tourists (see Fig. 1). Figure 1 shows the western temple tower as well as the main thoroughfares around the complex. These are the roads which ply thousands of diesel-operated vehicles around the heritage structure. Madurai’s global stature is intertwined with the temple, but currently faces environmental challenges, including urban pollution from unregulated vehicular emissions. Soot and black carbon haze emitted from vehicles leave their deleterious imprints on the gopurams and restoring them to their pristine glory is time-consuming as well as prohibitively expensive. If haze images can be reverse-engineered to reveal particle morphology, size distribution, and density, then non-invasive pollution assessment becomes feasible, even in congested and heritage-sensitive zones where physical sampling may not be possible. This study addresses the above issues and requires both modelling and experimentation, and the scheme of the paper is as follows:

(i) An assessment of how the Madurai region responded to increasing urbanisation over 1940–2050. During the 1940s, the vehicular density, heat stresses and pollution loads were a fraction of what they are now. With the economic liberalisation ushered in during the 1990s, private ownership of vehicles increased exponentially and with a surge in tourism the Madurai roads grappled with unsustainable vehicular densities. With the scourge of global warming, extreme events and heat stresses became the new norm and called for an assessment of how the region was expected to cope with in the coming years up to 2050—an RCP 8.5 year. This emission scenario is most likely to be a tipping point and calls for a quantification of the changes of the most telling indicators—Leaf Area Index (LAI), Temperature (T) distribution, Precipitation (PPT), Cloud cover (CC) fractions and Soil Moisture (SM) changes.
(ii) A one-off observational procedure to derive the Sauter Mean Radius (SMR) of the emitted particles from 4 stroke diesel engines under varying engine loads roughly proxying conditions along actual roads surrounding the complex.
(iii) Developing R-CNN algorithms to generate the SMRs from the extent of greyness of collected soot particles. The resulting particle-size distributions (DSD) for differing engine loads are crucially important to characterize the pressure calibrated sprinkler orifices for roadside spraying until such time when the surrounding roads only ferry non-polluting EVs.
(iv) AI ML based predictions for the anticipated LAI, T, SM, CC, PPT changes for the upcoming period 2025–2050. This crucial time range also is in sync with the Government of India’s time plan under its Smart City projects enabling major policy decisions to make regions around heritage structures sustainable by adopting non-polluting vehicles.
(v) The paper concludes with a discussion on wider implications with suggested well supported assessments. These include an assessment of the extent of soot deposition on the vulnerable west gopuram during two seasons for a business-as-usual case (i.e. with polluting vehicles) contrasted with the situation when Electric Vehicles (EVs) are in place.

Figure 1. Blender Image of Meenakshi Madurai Temple Complex showing temple Towers and circumscribing streets in the four cardinal directions. Note also the arrangement of main and arterial thoroughfares that fill to capacity with offending vehicles emitting soot and black carbon resulting in the soiling of the iconic heritage structure.

**Fig. 2:**
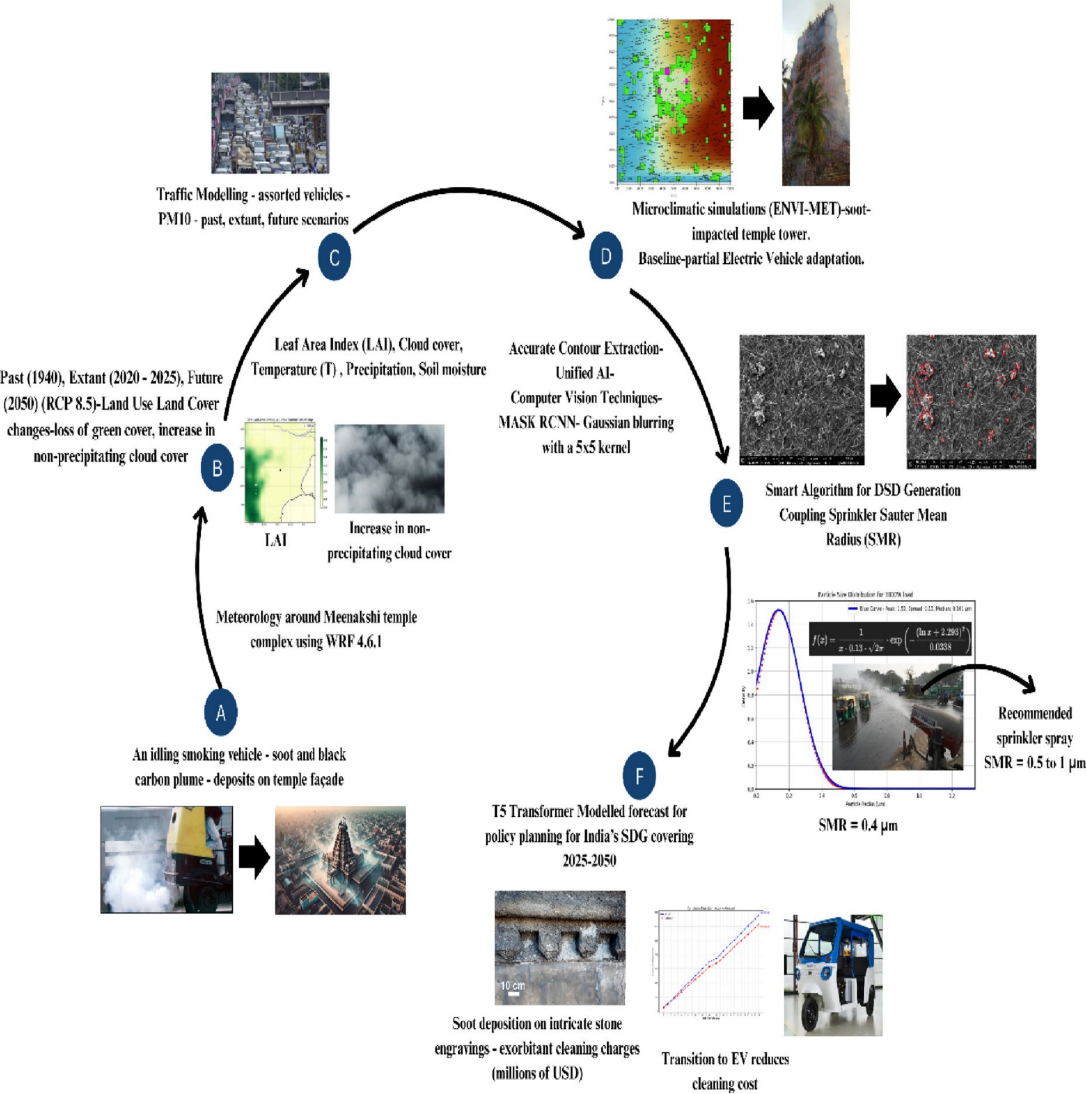
Schematic Layout for study overview. Emissions from malfunctioning vehicles emit soot and black carbon along main thoroughfares-North, East, West, South Chitrai Streets, followed by mixing in the lower boundary layer. This causes deleterious deposition on the temple façade. LAI and other LULC indicators—1940, 2021, 2050 (RCP8.5). Modelled PM2.5 and PM10 deposition fluxes with and without electric vehicle replacement. Morphological characterization of 4-stroke engine emissions at source with diverse size ranges. Grey-scale images initialize novel AI protocols to yield drop-size distributions. AI mediated forecasts with and without remediation at heritage site receptors-vastly improved cost savings and paybacks.

The concept diagram shown in Fig. 2 is a pictorial representation of all the procedural steps involved in this paper.

## Methodology

In this section we first describe the data used (“Data description”) for the Weather Research and Forecasting Model^2^. This is followed by a description of the Model Setup (“Model setup for weather research”). In “Experimental methods for soot characterization”, the procedural sequence in the experimental methods for the characterisation of the soot particles emanating from the IC (internal combustion) engines are described. The AI analysis follows in “AI analysis” which includes descriptions of the Transformer Model requirements.

### Data description

This study uses three distinct datasets to investigate atmospheric behaviour across various timelines, i.e., the past (1940), an extant (2021), and a future year (2050). For the year 2021, data was sourced from the NCEP FNL Operational Model Global Tropospheric Analyses (National Centres for Environmental Predictions), which provides global data on a daily basis from July 1999 onward. The data is obtained in GRIB2 format. For the projection of 2050, we sourced climate model outputs from the MESACLIP (MESOSCALE Atosphere-Ocean Interactions in Seasonal-to-Decadal Climate Prediction) dataset which is a 10-member ensemble of CESM (Community Earth System Model) high-resolution RCP 8.5 simulations. From this collection, atmospheric and land-data on a monthly basis were obtained with NetCDF. The baseline, data was sourced from the ECMWF (European Centre for Medium Range Wather Forecasts, 5 generation) ERA5 dataset to represent the pre-industrialisation year 1940, in NetCDF format matching the spatial and temporal requirements of this study.

### Model setup for weather research

The Weather Research and Forecasting (WRF) model was configured using the Advanced Research WRF (ARW) core for the mesoscale atmospheric simulation. The model domain covered 9.86° N and 78.10° E, utilizing a Mercator projection with a horizontal grid spacing of 3 km across a 100 × 100 grid run with a 6-h update interval^3^. Preprocessing steps were conducted through WRF Preprocessing System (WPS), involving geogrid, ungrib and metagrid utilities to transform GRIB2 meteorological inputs into intermediary and model-ready files.

The 3rd sequence of research methodology involved procedures that enabled the collection and characterisation of soot emissions from automobiles and is described below.

### Experimental methods for soot characterization

Diesel engines and their exhaust systems were prepared for the collection of soot and black carbon from the exhaust. These particles were collected from a four-stroke single-cylinder diesel engine with a power output of 5 HP (KIRLOSKAR/AV1). The exhaust system was configured to allow clear access to the exit pipe for particulate matter collection. Grade-1 micron filter paper (Supertek; material cellulose; pore size 11 microns; thickness 0.18 mm; retention fine but not extremely fine) was used to effectively capture the emitted particulate matter. The filter paper chosen was suitable for internal field emission scanning electron microscopy (FE-SEM) analysis and can withstand high temperatures during the particle collecting procedure^4,5^. The filter paper was carefully positioned directly over the exhaust pipe’s exit point. It was trimmed to an appropriate size and securely fixed in place using heat-resistant clips or adhesive materials. This ensured that the filter paper remained stable throughout the experiment, even under the influence of exhaust gas flow, and withstood vibrations. The diesel engine was run for a predetermined duration, allowing sufficient particulate matter to deposit on the filter paper. The operational conditions, such as engine speed and load, were maintained consistently to ensure the exhaust emissions represented normal engine behaviour. The duration of engine operation was carefully controlled to optimize the amount of particulate deposition in a controlled manner without overwhelming the filter paper. After this procedure, the particulate-laden filter paper was further analysed for morphological characterization using the FE-SEM facility^6^.

The subsequent experimental analysis involved the use of FE-SEM (Field Emission Scanning Electron Microscope), Thermo FEI QUANTA 250 FEG offering a high-resolution of 1.2 nanometres at 30 KV, along with Energy dispersive X-ray (EDX with sputter coating—Oxford Instrument^7^. Since the collected particles were largely composed of carbons, a non-conducting substance, a subsequent procedure involving a pre-coating of gold was used for the analysis. Engine load 0 corresponds to vehicle idling conditions—a common occurrence in the congested city of Madurai. Typical waiting times along roads opposite the west tower is of the order of several minutes. This allows the build-up of residual pollution comprising PM 2.5 and PM 10 which hovers over the region for several days even when the wind direction changes. As the engine load increases the particle number concentrations dramatically^8,9^.

The fourth stage of the research methodology involved a comprehensive analysis involving AI analysis to yield reliable environmental projections and this is described below.

### AI analysis

**Fig. 3:**
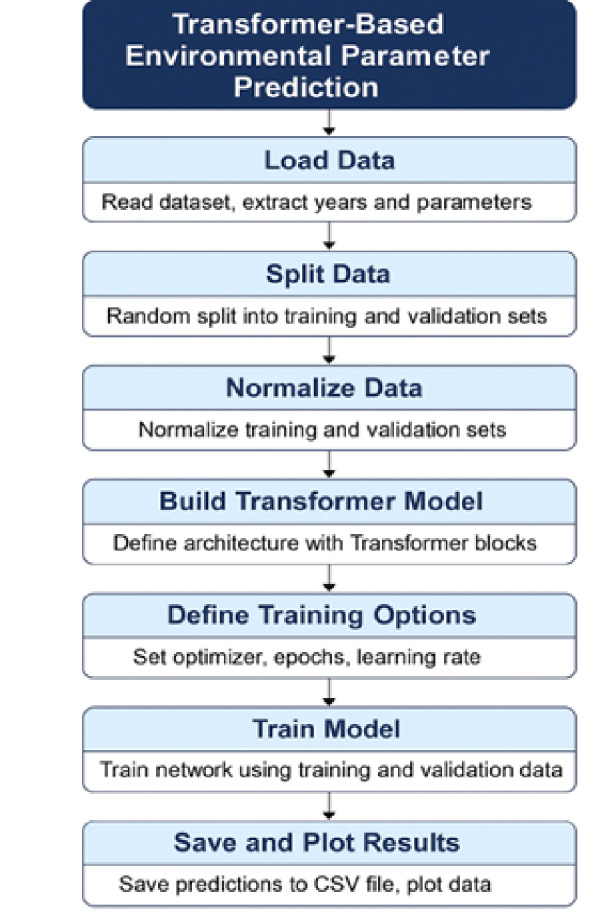
Transformer based prediction process customised to yield specified environmental indicators.

In this section the detailed parameters and assumptions in the AI modelling are first described and thereafter workflow diagrams are presented. A pseudocode explaining the flow of the algorithmic python tools and libraries is shown in Fig. 3 and the procedural sections are self-explanatory as described in the sequential command lines shown.

**Fig. 4:**
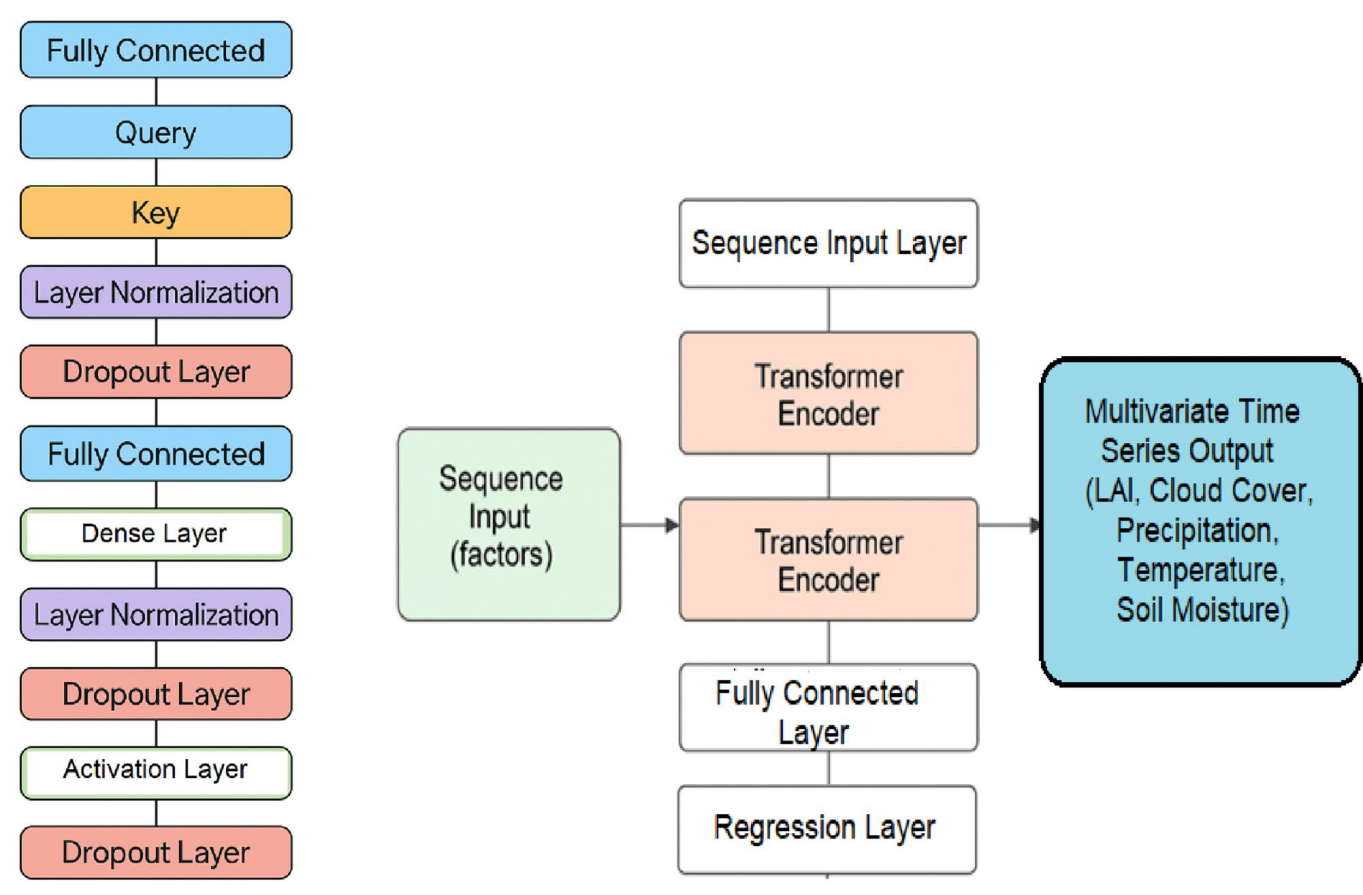
(**a**) Architecture for proposed transformer based model (**b**) Transformer encoder block diagram.

A transformer application is used for the prediction model involving large datasets necessary for Land Use Land Cover (LULC) categorization as well as for climate modelling. The key attributes are indicated in Figs. 3 and 4 shown below.

An AI framework was implemented using MATLAB 2024a, integrating deep learning and transformer modules for environmental parameter prediction, whilst the dataset consisted of environmental feature samples, split into 70% training, 15% validation, and 15% testing sets using randomized indices. For the Normalization, a Min–max normalization was applied using a custom MATLAB function (the code is made available in the supplementary data attached) to ensure consistent scaling across the training and validation data. The model consisted of the following attributes − 5 Transformer layers; 4 Heads; Feature dimension (dModel) 64; Feedforward dimension (dff): 128; Dropout rate 0.1; Epochs 500; Optimizer: Adam (InitialLearnRate = 0.001); Mini-batch size 3. Additional details are shown explicitly in Table 1.

These hyperparameters were selected after multiple pilot runs to achieve stable convergence with minimal validation loss.

A MATLAB prediction code (provided as supplementary data) achieves a complete process of data normalization along with model training, validation, and future value prediction capability.

Figure 3 shows a flow chart to build, train and interpret the transformer model, whilst Fig. 4 shows the layer-wise architecture.

Transformer-based models can now be straightforwardly interpreted with accuracy for multivariate time series forecasting^10–16^. Figure 8 shows its architecture adopted in this study and uses past sequences of environmental factors. It incorporates sequential inputs of the environmental parameters, LAI, CC, TP, T, and SM through a sequence input layer. The data is then processed by two stacked Transformer Encoder layers, which learn complex temporal patterns and dependencies. The output is passed through a fully connected layer and a regression layer to predict multiple future environmental parameters.

The model has proven performance attributes vis-a-vis several evaluation metrics including accuracy, compares favourably with other established models - Multi Patch Former and PRFormer. Reassuringly, it achieves the lowest mean squared error (MSE) and mean absolute error (MAE) seamlessly with benchmarked datasets. The flow diagram describes this transformer-based model for multivariate time series prediction. The model processes sequence input factors through stacked transformer encoder layers, followed by a fully connected layer and a regression layer, to generate outputs for multiple variables such as LAI, CC, TP, T and SM.

Training data was sourced from the years 1940–2024. The predicted results were validated against the actual data and the RMSE yielded high prediction accuracy with an average RMSE of 0.02.

Any Transformer model’s robustness is tied down to its hyperparameters, which have to be very carefully selected so that it efficiently captures the loss landscape and avoids any sub-optimal minima.

The Table 1 below summarizes itemises the key attributes of the Transformer Model.

**Table 1:** Transformer model summary indicating the hyperparameters in the first column with the description of each one of them.

Parameter	Value	Description
Input dimension	1	Single input feature (Year) via sequence Input Layer (1)
Output dimension	5	Predicts 5 parameters: LAI, Cloud Cover, Precipitation, Temperature, Soil Moisture
Number of transformer blocks	2	Two repeated custom transformer blocks for feature transformation
Model dimension	64	Size of the feature space after the embedding layer
Number of attention heads	4	Specified but not explicitly implemented as true multi–Head Attention (approximation only)
Feedforward dimension	128	Size of hidden layer inside the feedforward network in each transformer block
Dropout rate	0.1	Used in dropout layers for regularization
Activation function	ReLU	Non-linearity in feedforward layers (ReLU layer)
Normalization	Layer norm (2 per block)	Applied after attention and feedforward stages
Training epochs	500	Maximum number of training epochs
Batch size	3	Small mini-batch size for gradient updates
Optimizer	Adam	Adaptive optimizer used in training options
Initial learning rate	0.001	Starting learning rate for optimization
Loss function	MSE (mean squared error)	Default regression loss in regression layer

## Results and discussions

As indicated in “Data description”, the state-of-the-art weather and research forecasting model (WRF) Version 4.6.1^2^ was used to contrast a pre-economic liberalisation year (1940) with an extant year (2021) and a future high emission RCP 8.5 scenario year (2050) for ascertaining the loss of vegetative cover (LAI) and other environmental indicators around the temple complex. A single grid setup was used with grid dimensions – dx, dy, 3 km × 3 km, and time step of 6 h and implemented a simple 3-class scheme for cloud water, rain, and ice along with the Kain–Fritsch scheme and used the standard USGS land use categories.

The WRF yielded patterns of LULC Changes with increased urbanisation, and changes in CC, T and SM, and a widely used microclimatic model, ENVI-met version 5.7.1^17,18^, was used to assess PM10 deposition on a receptor, the west gopuram in this instance. The model is compatible with GIS data and includes visualisation capabilities supporting urban planning initiatives seeking to balance heritage conservation with sustainable urban development. Some relevant Indian studies done for a heritage facade for the city of Lucknow validates the choice of the microclimate model^19–22^ ENVI met was run over 2 months (February with SE flow and July with a NW flow) corresponding to two seasons when the depositions are significant. It was used to simulate two situations—(i) a business-as-usual case when diesel vehicles plied unrestricted i.e. the baseline case and (ii) the case with a replacement of the diesel vehicles with EVs.

As indicated in the introduction, it was necessary to first capture and then characterise soot and black carbon particles emanating from 4 stroke diesel engines—the most common plying vehicle over the region.

Amidst unbridled urbanization, Madurai’s Land Use Land Cover (LULC) profile has changed for the worse^23^, especially around the Meenakshi Amman Temple. Vegetated lands and open water bodies that once shielded the temple have been replaced by urban expansion, dense road networks, and impervious surfaces with concrete and asphalt (see Fig. 1). Streets surrounding the temple area such as North Chitrai Street, South Chitrai Street, and other arterial routes have transitioned into high-traffic zones, drastically increasing soot and black carbon emissions. The loss of green spaces has subdued the natural ability of foliage to scavenge pollutants from the air. LULC shifts heighten particulate matter (PM10) deposition patterns during dry seasons, intensifying discoloration and erosion of the temple’s facades. Their submicron size and prolonged atmospheric residence time (up to 10 days)^8,21^ allows them to disperse and adhere to porous stone surfaces, catalysing chemical reactions. Increased aerosol concentrations suppress droplet coalescence to initiate the processes of cloud auto-conversion and accretion. This causes a suppression of precipitation which would have partially cleansed the pollution pall.

### Temperature extremes, leaf area Index, cloud cover and precipitation pattern changes

**Fig. 5:**
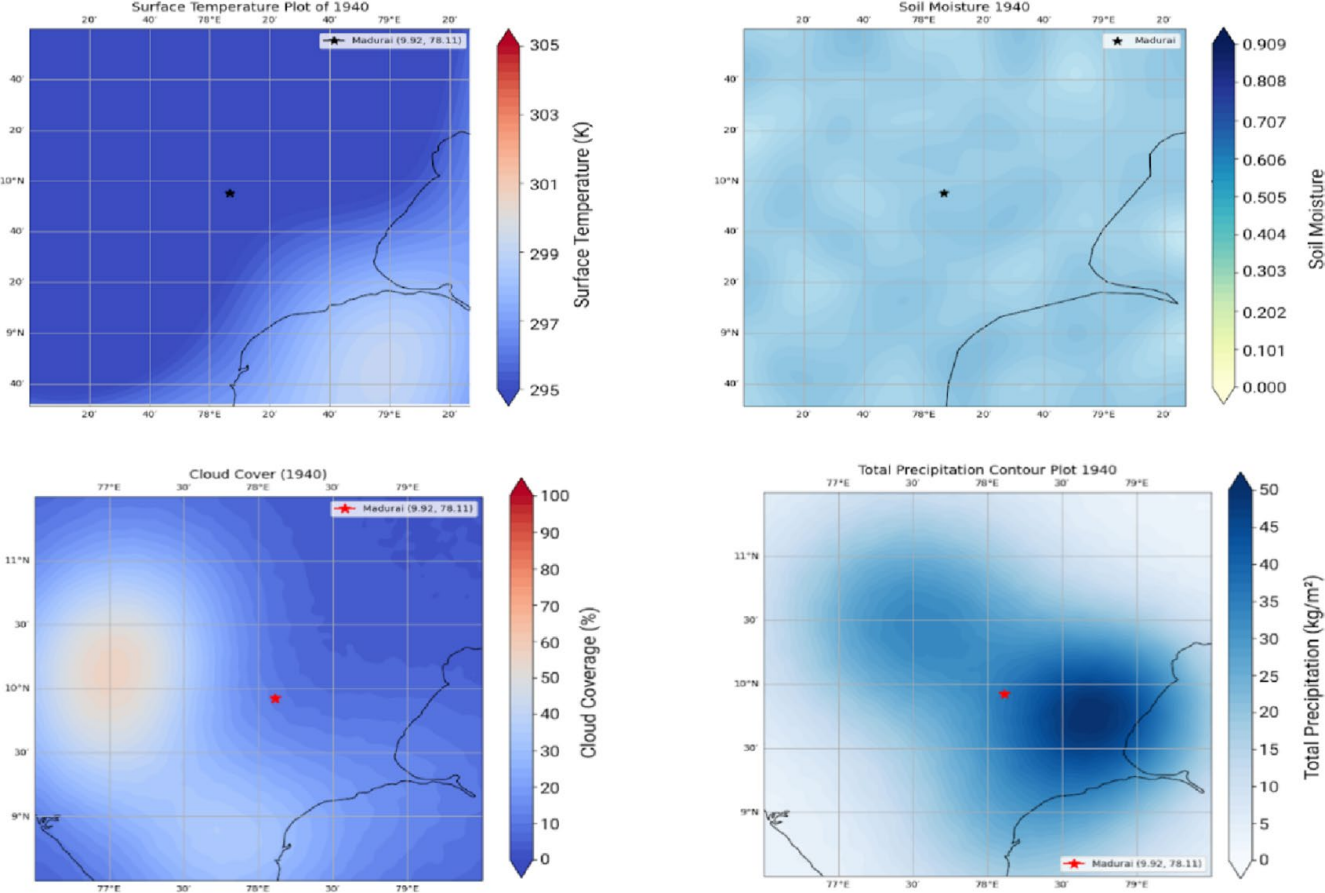
1940 temperature distribution corresponding to pre-economic liberalization (top-left). One notices the temperatures are in the range of 295–297 K in the vicinity of Madurai. 1940 Cloud Cover, low to moderate cloud cover during the month of February, after the retreat of the northeast monsoon. (bottom-left). 1940 Total Precipitation ranges between 25–30 kg/m^3^ during this month. (bottom-right). 1940 Soil Moisture directly linked to the above three parameters indicate reasonable wetness of 0.55. (top-right). Data sourced from ECMWF Reanalysis v5 (ERA5) Source. ECMWF Reanalysis v5 (ERA5) Dataset.

**Fig. 6:**
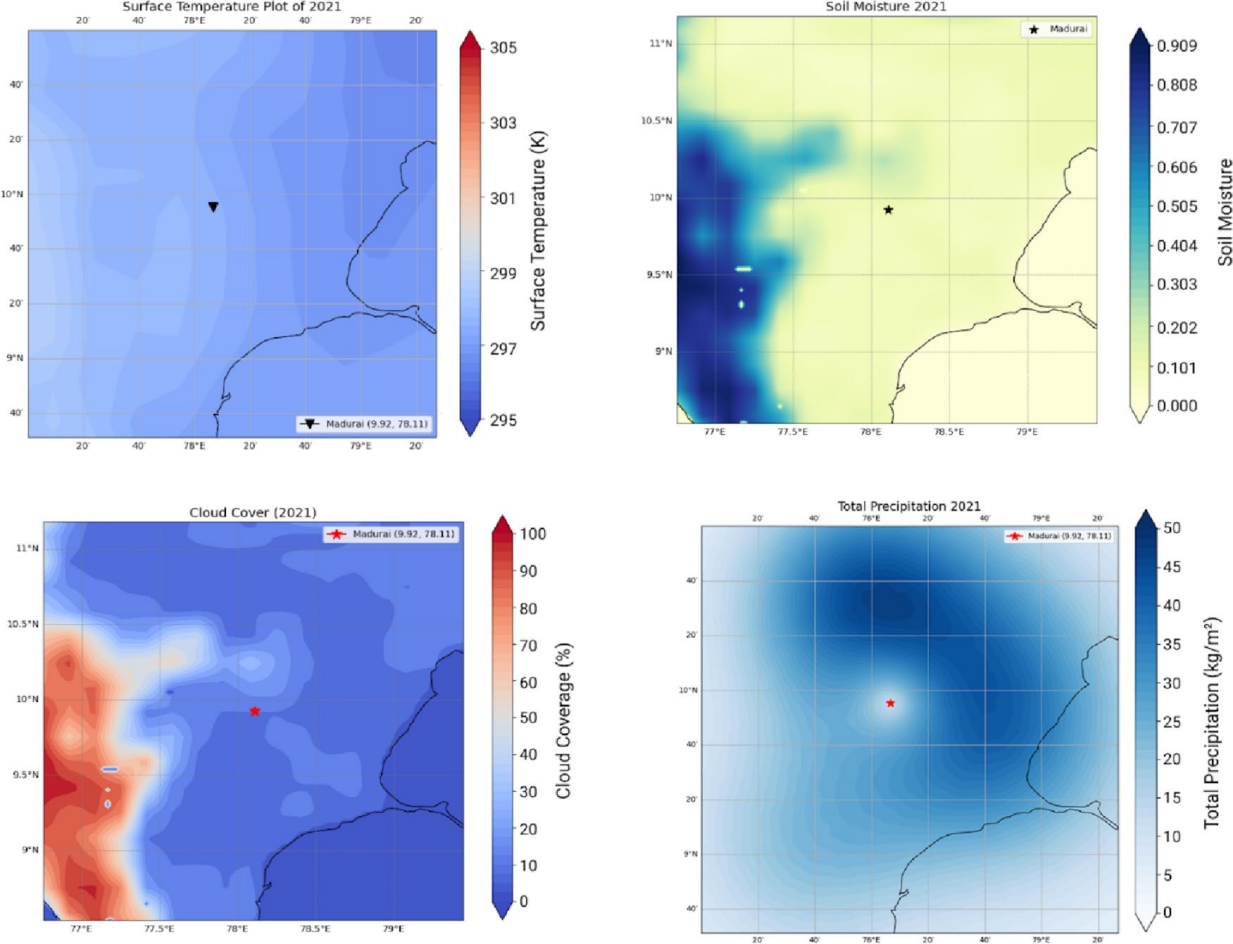
A representative post-economic liberalization year (2021—temperature distribution around the heritage site. One notices the temperatures are in the range of 297.6–299.8 in the vicinity of Madurai, almost 2 K higher than what obtains during 1940 (top-left). There is a 63.6% increase in Cloud Cover compared to 1940-year values are in the moderate range during the month of February, after the retreat of the northeast monsoon (bottom-left). Total Precipitation ranges between 5–10 kg/m^3^ during this month, a 68.3% decrease compared to the values obtained for 1940 (bottom-right). Soil Moisture directly linked to the above three parameters indicate lower wetness. A decrease of 13.4% is observed compared to 1940 (top-right).

**Fig. 7:**
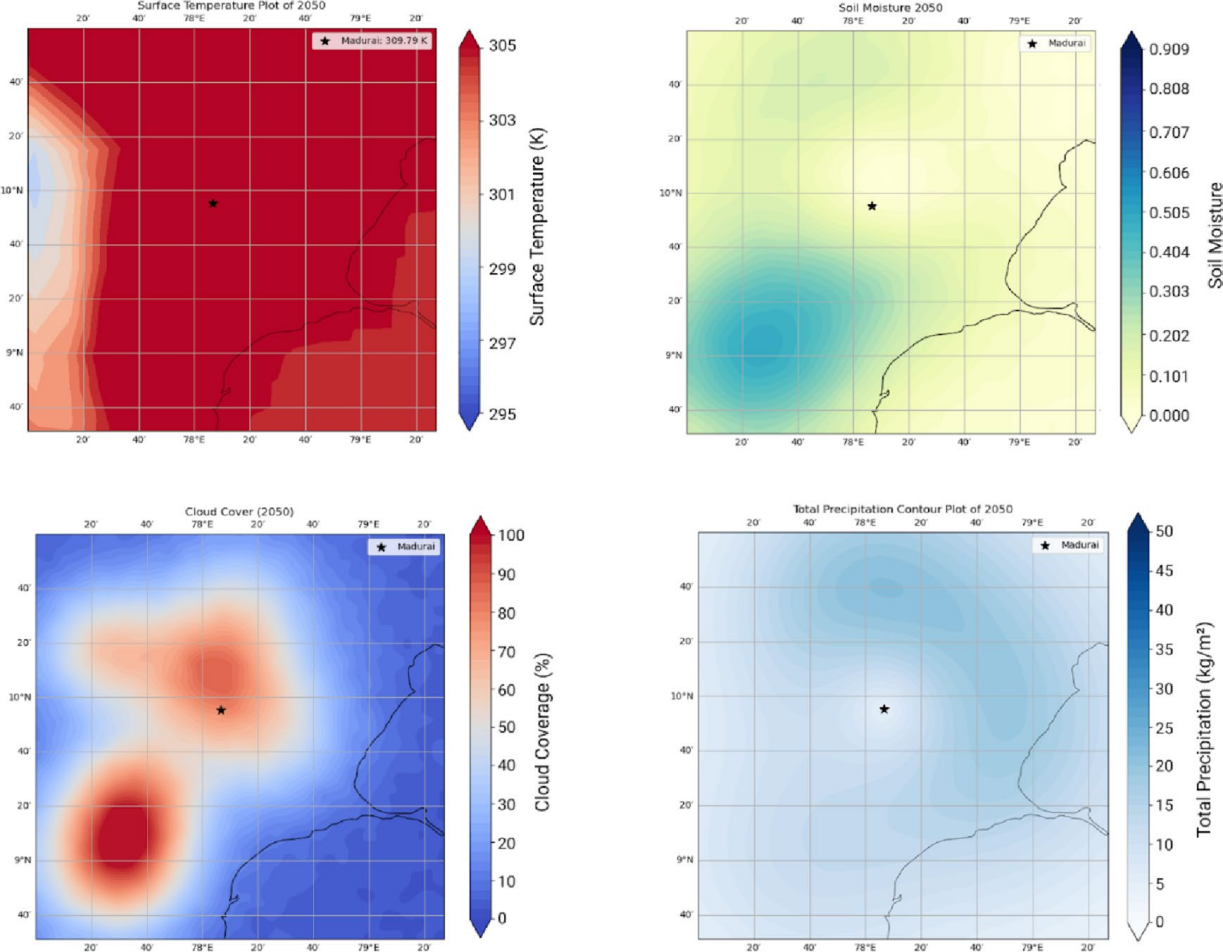
High emissions RCP8.5 scenario 2050. Temperature distribution around the heritage site. One notices the temperatures are in the range of 304–305 K in the vicinity of Madurai, 4 K higher than the case shown for 2021 (top-left). Significant increase in Cloud Cover in the range of 57–65, 44.4% higher than what obtains in 2021 (bottom-left). Increasing pollution adds more particles to the atmosphere competing with a limited amount of moisture resulting in suppressed cloud droplet diameters lower than the critical threshold of 20 microns resulting in reduced collision and coalescence processes. This implies a concomitant decrease in precipitation (bottom-right). The results shown in the year 2050 are indicative of high temperatures coupled with decreased precipitation resulting in a drastic loss of moisture from the soil to the tune of 46.2% as compared to that of the year 2021 (top-right). Data sourced from MESACLIP: A 10-Member Ensemble of CESM HR RCP 8.5 Simulations Source: MESACLIP Dataset.

The WRF yielded LAI Changes for over 3 months (Feb, characteristic of a relatively cool month, June, the hottest month, and November a monsoon month during 2021). The outputs for 2021 are compared with 1940 (pre independence low emissions case) against the RCP 8.5 high emissions case. A quantification of LAI over these timelines is linked to ground temperature distributions, the cloud cover and soil moisture. We show 3 sets of results. Figure 5 shows Surface Temperature, Cloud Cover, Soil Moisture and Total Precipitation for the pre-independence year 1940. Figures 6 and 7 respectively corresponds to these very indicators the years 2021 and 2050.

\

**Fig. 8:**
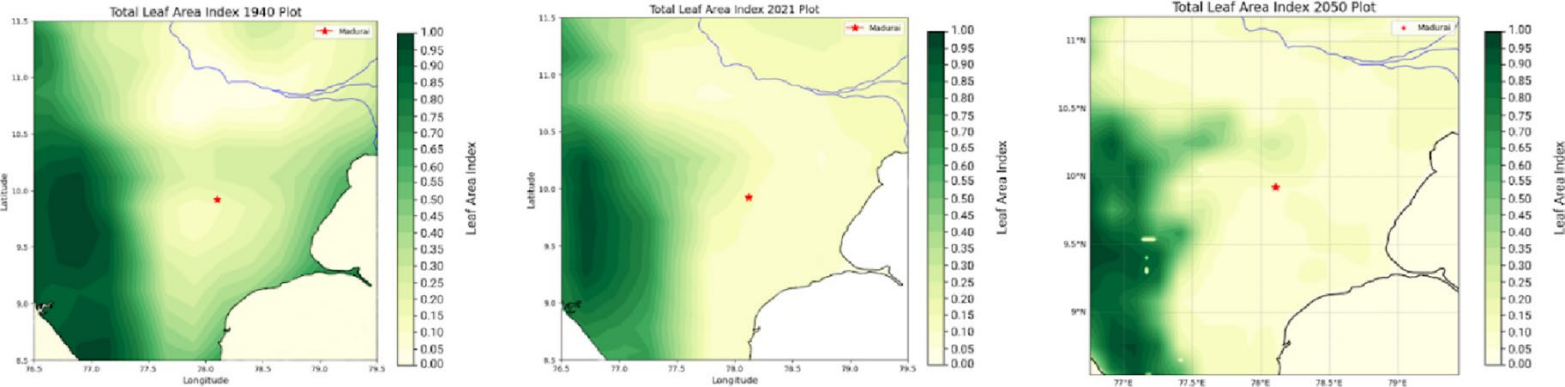
(**a**–**c**) Leaf Area Index changes during 1940, 2021–2050.

One notices surface temperatures ranging from (295–297 K) over the Madurai region (encircled in Red). The soil moisture, cloud cover and total precipitation -all affect the Leaf Area Index (LAI) and one observes that the patterns of these indices progressively worsen during 2021—a year well entrenched within India’s post liberalisation era. The freed-up economy promoted a boom in infrastructure development and enabled home ownership rapidly over this region and from a perusal of Fig. 1, one can appreciate the exceedingly high density of built-up areas contributing to the urban heat island effect, increasing surface temperatures and causing a loss of soil moisture^24^. Owing to an increase in vehicular traffic, with malfunctioning diesel vehicles spewing out copious amounts of soot and black carbon, there is a further aggravation related to the high airborne residence times of these small (< 1 μm) particles adding to the high PM load. Since they hover around for so long, these originally hydrophobic particles accrete soluble sulphates and nitrates to become cloud condensation nuclei. However, owing to the high number concentrations and a limited amount of atmospheric moisture, their growth rates get slowed down and a majority of them are unable to cross the Kohler barrier so that runaway growth of cloud droplets is completely inhibited. Since these cloud droplets are very small, they are unable to cross the crucial threshold of a 20-µm radius to promote collision and coalescence crucial to rain formation^25–32,33^ and although they cause an increase in cloud cover, they are unable to precipitate. This explains the progressive increase in CC and decrease in precipitation in Fig. 6 during 2021. This trend is further exacerbated (see Fig. 7) for the high emission year 2050. With a progressive loss of precipitation and soil moisture, one finds a corresponding decrease in the LAI as one proceeds from 1940, to 2021 and thence to 2050. This marked and progressive loss of the LAI (see Fig. 8) indicates that botanical solutions offered by vegetation to cleanse particulate pollution are curtailed^34–36^. This brings us to consider the second more powerful alternative; i.e.; wet scavenging mediated by carefully designed sprinklers suitably calibrated so that they release droplets in the right range of SMR of the released spray- they should provide the maximum spray mist aerial coverage of the right median diameters with substantial number densities to be able to efficiently scavenge soot and black carbon. Efficient scavenging depends not only on the drop size distribution (DSD) of the aerosol particles; they also depend on the size of the collector droplets released from sprinklers. Since we are dealing with large amounts of submicron particles, additional procedures in the form of pressurised spraying will have to be considered to achieve a successful washout. For all these reasons it is crucially important to get a handle on the size distribution of the polluted aerosol particles in a quick and efficient manner to proceed further with any amelioration measures^37–39^).

Figure 8 shows the changes observed in the distribution of the Leaf Area Index (LAI) over the region with Madurai indicated as a dot withing a red circle for the years 1940, 2021 and 2050. The combined impact of increased temperatures suppressed precipitation, and loss of soil moisture is best captured by studying changes in the LAI—a progressive loss of LAI suppresses the natural trapping of particulate matter by vegetated surfaces^34^. This was also studied over the same periods.

The receptor site is indicated by a red star. Data sourced from ECMWF Reanalysis v5 (ERA5) Source: ECMWF Reanalysis v5 (ERA5) Dataset.

One notes drastic reduction in LAI (48.3%) from 2021 onwards peaking to much lower values during 2050 for a vast swathe, both on the left and the right of the Madurai region. During 1940 (left panel) shows high LAI values on either side of the receptor. This feature is missing in later years. LAI values only diminished along swathes on the left. Data sourced 2021 from WRF outputs; 1940, 2050 from MESACLIP: A 10-Member Ensemble of CESM HR RCP 8.5 Simulations Source: MESACLIP Dataset.

#### Soot and BC loads from diesel vehicles over the Madurai region

Since 1940, vehicular density has risen exponentially, fuelled by economic liberalization, and industrial growth. However, even until the present period there has been inadequate investment in sustainable public transport. Traffic congestion levels on arterial roads such as North Chitrai Street, South Chitrai Street, East Masi, and West Masi Streets (see Fig. 1) are among the highest in Madurai, contributing to heavy emissions of soot and black carbon. Diesel-powered vehicles, which constitute the majority of traffic, emit up to 60% of PM2.5 and PM10 pollutants in the area. In the absence of greener transport strategies, the imprint of particulate deposition onto exposed surfaces and over the gopurams of the Meenakshi are easily visualised even today and will rise by 2050. The transition to green technologies, specifically electric vehicles (EVs), presents a promising solution but is progressing at a slow pace. A better strategy would be to first quantify haze particle size distributions around the temple complex in a robust and quick manner so that customised sprinklers can be deployed at vantage points with calibrated spray nozzles which would release collector droplets of the right size as discussed earlier. It is shown here how a transformer model output can provide this crucial information with ease for use in a foolproof engineered wet scavenging operation. This is discussed below.

#### A novel protocol for particle detection, radius estimation and drop size distribution characterization based on mask R-CNN and computer vision techniques

Since, the particulate number loads are crucial for the present study, a novel AI ML method is described that yields particle-size distribution curves as a function of visual greyness captured on camera. Size segregation is important because, the smallest (less than 1 μm radius) have a high residence time of the order of 7–10 days^8^ and contribute significantly to the residual pollution, so that even when the wind direction changes, the residual particles, will be able to dry deposit on the temple façade slowly over time. In contrast, the larger ones, over time, accumulate enough airborne sulphate to serve as cloud condensation nuclei. FE-SEM is adopted to visually capture particle sizes from 4 stroke diesel engines at various engine loads as well as zero load idling conditions.

The python script for processing and analysing the FESEM generated morphological images of the soot and black carbon trapped in the filter paper facilitates a quantitative analysis for the detection, characterization, and visualization of these particulates. The core workflow is structured to ensure reproducibility, scalability, and precision in particle size estimation, which is critical for choosing an optimal Sauter Mean Radius (SMR) for the most efficient wet scavenging.

The script first imports the essential scientific computing libraries: OpenCV (cv2) for high-performance image processing -NumPy for efficient array-based mathematical operations, and Matplotlib for result visualization. The analysis begins by loading both a reference image and a test image. The reference image, typically containing well-defined particulate structures, is used to calibrate thresholding parameters, while the test image is the primary subject for particle analysis. Both images are converted to grayscale to standardize the data and reduce computational complexity, as colour information is not pertinent to the detection of carbonaceous particulates. Preprocessing is performed using Gaussian blurring with a 5 × 5 kernel, which attenuates high-frequency noise and enhances the robustness of subsequent thresholding. A fixed-intensity global threshold (set at 200) is then applied to both the reference and test images, converting them into binary masks where particles are represented as high-intensity (white) regions. This binarization is critical for accurate contour extraction, as it delineates particle boundaries against the background.

To mitigate the influence of non-sample artifacts, such as scale bars or interface overlays commonly present in microscopy outputs, the script programmatically zeros out the bottom 100 pixels of the test image’s binary mask. This targeted artifact removal enhances the specificity of the particle detection process. Contour detection is executed using OpenCV’s find contours function with the RETR_EXTERNAL retrieval mode and CHAIN_APPROX_SIMPLE contour approximation. This configuration ensures that only the external boundaries of discrete particles are extracted, optimizing computational efficiency and reducing false positives from internal noise or holes. For each detected contour, the script calculates the enclosed area using the countour Area function from the opencv2 library (https://opencv.org). Only contours with non-zero area are retained for further analysis, thereby filtering out spurious detections. A critical technical aspect is the conversion from pixel-based measurements to physical units. The script employs a calibration factor (0.1953 μm/pixel), derived from the imaging system’s scale bar, to convert the computed contour areas into real-world dimensions. Particle radii are estimated by modelling each contour as a circle of equivalent area. The resulting radii are then scaled to micrometres, enabling direct comparison.

For visualization, the script overlays the valid contours onto a copy of the original test image using a distinct colour (blue), providing immediate visual confirmation of the segmentation results. The annotated image is saved for documentation and further analysis. Additionally, the script outputs the total number of detected particles and reports the radius of each particle in micrometres, formatted for ease of interpretation and downstream statistical analysis.

This semi-automated pipeline offers a robust, non-destructive methodology for quantifying particle size distributions from microscopy images. Its design ensures high throughput, reproducibility, and adaptability to various particulate systems, making it suitable for both routine laboratory analysis and the generation of labelled datasets for machine learning applications such as Mask R-CNN-based segmentation. The technical rigor of the approach, including explicit calibration, artifact mitigation, and precise geometric modelling, underpins its reliability for scientific research and industrial quality control.

Table 2 shows the captured visual intensity, the morphology of the captured particles and the estimated Drop Size Distribution (DSD) in the three columns.

**Table 2:**
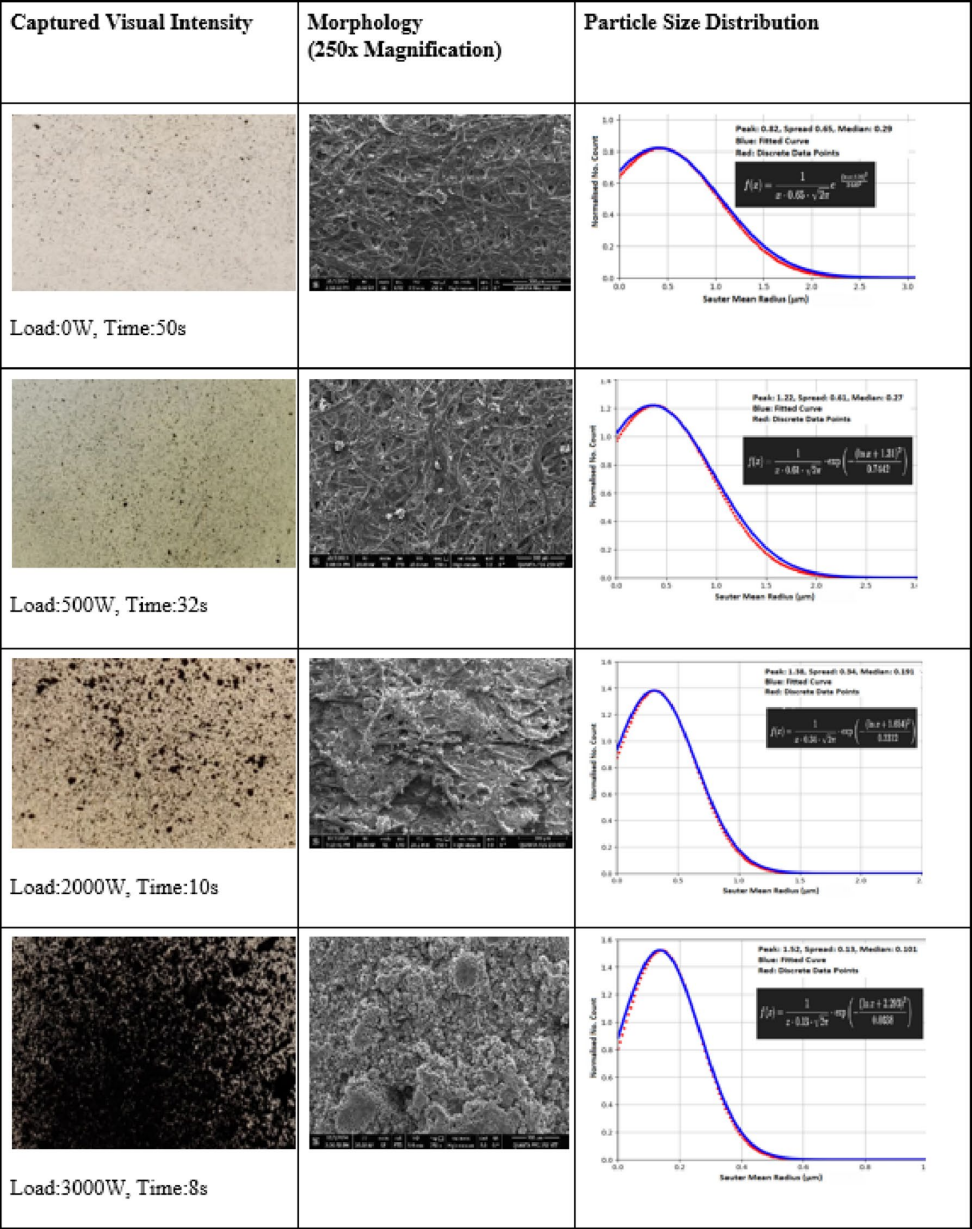
Procedural sequences for characterising particle size distribution using FE-SEM.

It is observed that with increasing engine loads, the extent of greyness increases significantly, as is evident from the Table 2—even during idling conditions the particle counts are high and increase several times at full load. There is a progressive decrease in the SMR with increasing loads and most significantly, the fraction of sub-micron particles increases when one contrasts the idling condition case with the full load case. This has a significant environmental consequence as described earlier and is tied down to a lowering of received rainfall, an increase in CC and the drying up of the soil moisture causing a decrease in the LAI. What follows is a description of these attributes over an extended period.

#### Transformer model predictions contrasting key environmental indicators-LAI, T, CC, soil moisture during the time slot 1940–2060

The year 1940 was chosen as a baseline case because around that time the Meenakshi Temple was surrounded by pristine settings with minimum vehicular emissions in contrast to the year 2020 when there was a substantial increase—in the former year whilst the density per kilometre were in their tens, in the latter they increased to a staggering value of several hundreds. (2020 (Ministry of Road Transport and Highways, Government of India, as reported by CEIC Data. 1940 estimate (https://www.ceicdata.com/en): Based on historical context from the Ministry of Statistics and Programme Implementation (MoSPI) and national vehicle registration trends.)

We first show 1D yearly averaged plot for the predicted trends for these parameters to have an overall handle on the rate of increase/decrease over this time period. This is achieved through MATLAB coding (available in supplementary data).

The code first web scraped data for LAI, Temperature, Soil moisture and Cloud Cover over the period 1940–2024 (https://cds.climate.copernicus.eu) and thereafter the sequence of steps indicated below was followed.

**Fig. 9:**
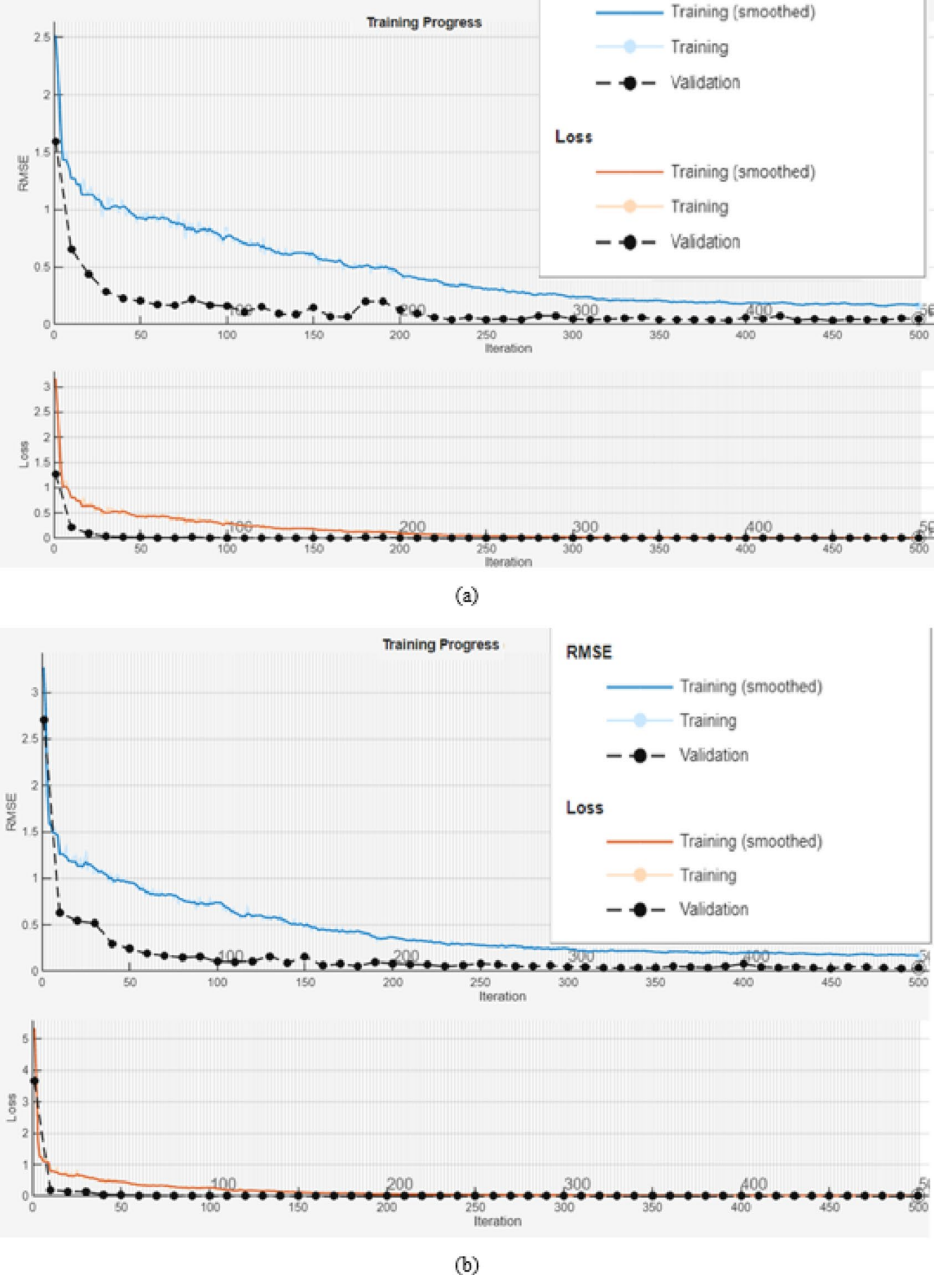
Stability progression of model (**a**) Training and validation (actual case), (**b**) Training and validation (contrast case).

The top panel of Fig. 9 shows that the model’s prediction stabilises and converges the after the 250th iteration. The bottom panel shows that the evaluation metric- loss is at its lowest after the 200th iteration.

As shown in Fig. 9, although RMSE Training and Loss start high (~ 2.5) they quickly decrease and smoothen by the 500 iterations. The consistent downward trend indicates that the model quickly learns and trains data confirming convergence and rapid improvement even at the early stages of training. The model remains flat and has a near zero RSME (~ 0.04E) for the rest of the training.

**Fig. 10:**
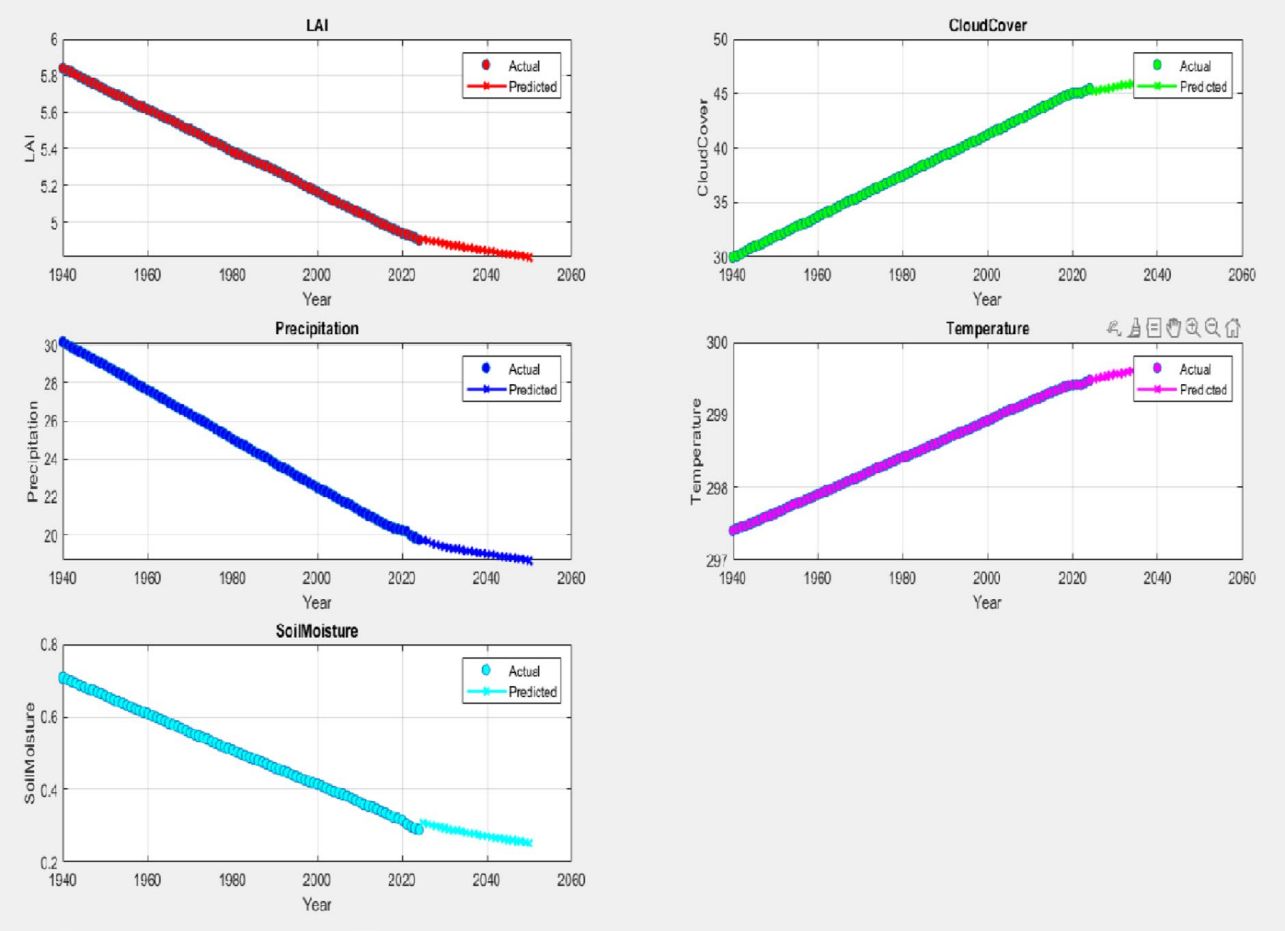
Modelled trends for LAI, CC, PPT, T and SM over the time period 1940–2050. 1940 is representative of the pre industrialised period of the country, whilst the 2020s represent a time slot over a fully deregulated economy accelerating an increase in urban built environments; 2050 is representative of the high emission RCP8.5 year.

The Final Validation RMSE at 0.038807 shows excellent generalization devoid of both overfitting and under fitting. Having demonstrated the robustness of the model we now look at the yearly trends of 5 environmental parameters relevant to this study-LAI, T, CC, PPT and Soil moisture. These are shown Fig. 10.

**Fig. 11:**
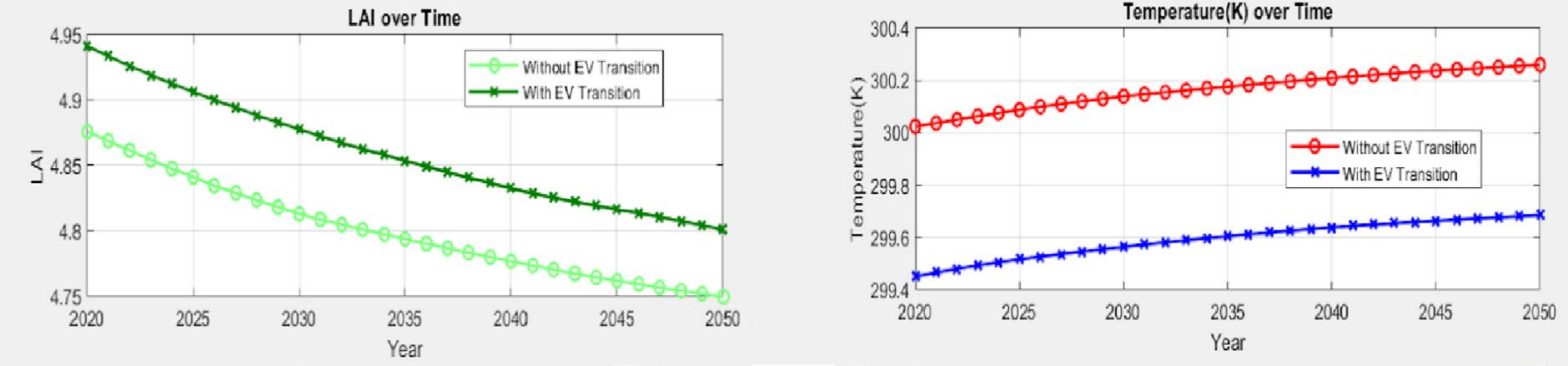
Transformer model predictions for key environmental parameters in 1D for temperature (K) and LAI. Note significant improvement with EV transition.

The most significant observation in all these figures is that there is a clear change of slope in the early 20s testifying that with the Madurai region getting completely built up, the rapid changes in the microclimate over the earlier period (1940–2000) was arrested. The increase/decrease over this plateaued up period happens from greenhouse gas build up alone. It is anticipated that the Madurai region will continue to experience increasing heat stresses over the period 2025 to 2050 drying up soil moisture with a suppression of precipitation with increasing PM pollution loads as discussed earlier. Overall, the LAI worryingly decreases over this period suggesting that the natural deposition of soot and black carbon on vegetation is much curtailed. Interestingly, the rate of increase of CC and T is almost exactly matched by the rate of decrease of precipitation, soil moisture and LAI. The redeeming feature of this conundrum is that with the introduction of EVs it is possible to further quantify temperature and LAI changes from the point of their introduction (2020) right up to the RCP8.5 year of 2050. This is shown below. One may notice that there is a 0.6 degree C temperature decrease with a 50% replacement. Concomitantly there is an overall increase in LAI as well (Fig. 11).

The fact that the predictions closely track the actual data with hardly any deviation lends confidence to explore in detail as to what might be obtained within a 2D framework. These 1d results suggest that the model understands precipitation reduction patterns, possibly influenced by climate change and regional aridity. The more subtle temperature trend shows first a rapid increase and thereafter a slower increase and yields a high predictive accuracy for temperature- the Madurai region has over the years experienced several heat surge days (Refs) and it is redeeming that the model is sensitive to small-scale thermal trends across decades. Soil Moisture trend also decreases faster over 1940–2020 and thereafter the decrease significantly slows down the decreasing trend and there is always a tight alignment with actual values showing that the prediction model effectively also captures long-term soil degradation, clearly linked to decreasing rainfall and vegetation.

The robustness of this prediction enables quick alerting predictions well into the future i.e.: 2030 to 2060 should this become necessary so that sufficient lead time and crucial foreknowledge are accessible to urban planners. The predictions show clear anti-correlations between LAI decrease and T increase. However, this rate of increase/decrease slows down from 2020 to 2060. The very rapid increase/decrease during 1940–2020 can be attributed to the surge of urbanisation that happened in an unregulated way which included unbridled private ownership of vehicles. The extent of concretisation and a near saturation urban density largely remained at a plateau (see Fig. 1) unlike in the earlier time slot when the urban density progressively increased,

The above results show that the Transformer model performs exceptionally well across all five parameters with validation plots showing tight coupling between predicted and actual values, even over multiple decades. The setup (with deeper layers and validation monitoring) led to a well-regularized model capable of multiple applications including forecasts over 2025–2050 projections and yield projection maps in 2D corresponding to what was shown earlier from the WRF model outputs (see Figs. 5, 6, 7 and 8).

#### 2D forecasts

**Fig. 12:**
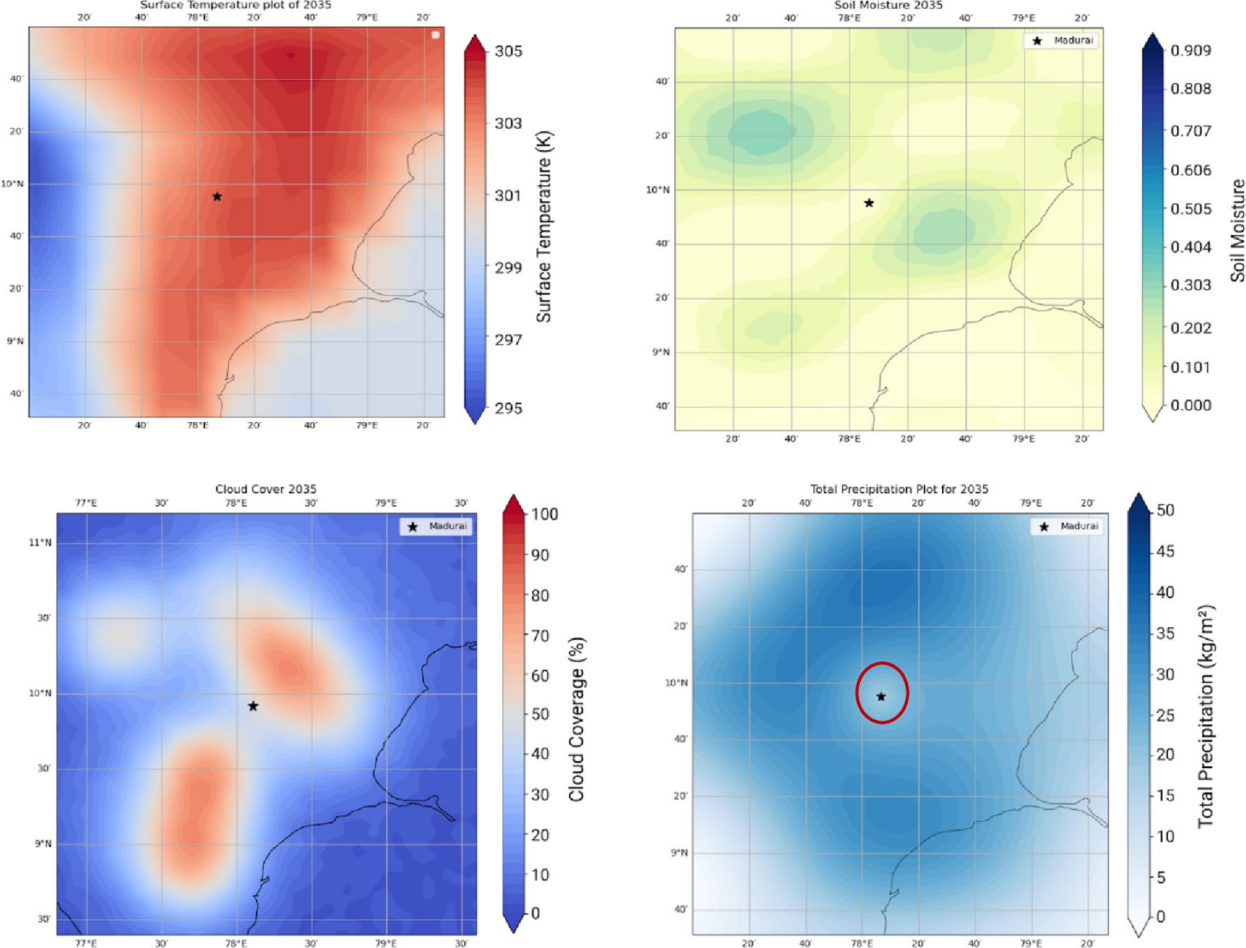
Validation of transformer model with a transition from 1D to 2D for T, TPP, CC, and SM. Almost Identical values obtained for all 4 environmental parameters.

The Madurai Meenakshi region has now entered a phase of almost saturated urban density, and one must explore warming associated with the coming decades up to the RCP8.5 high emission scenario. This exploration must also provide a framework for urban planners and decision makers to be able to use predicted forecasts with ease and simplicity, especially by personnel not experts in fluid mechanics. The WRF model requires an in-depth understanding of environmental fluid mechanics is hugely data intensive and must be run on powerful computers. A Transformer model, on the other hand yields, as we have just seen, very accurate predictions cheaply and quickly and is not at all resource intensives. It is anticipated that the municipal planning authorities dealing with the microclimate of the Meenakshi temple will need 2D maps of the LAI, T, CC, PPT and soil moisture for leveraging planning and development strategies for the future. This is shown in Fig. 12 below. A remarkable robustness of predictions is maintained even after transition to 2D. For example, from Fig. 12 2D plot, at the point of interest i.e.: the Meenakshi Amman temple, the soil moisture is the order of 0.27-this is identical to the value yielded by the Transformer in 1D. If city planners were to consider alterations in rerouting traffic or seek botanical solutions by planting more trees, they would look for locales far remote from the temple complex. This confirmation provides a justification for the use of 2D plots i.e.: As shown, there are 3 SM contours of values ranging between 0.18 and 0.24 between the latitude (78°E- 20) and longitude (40 –10°N) covering the intersection of the East and the North Chitrai streets surrounding the Meenakshi Amman Temple.

## Limitations and future work

This paper shows how unregulated vehicular pollution characterised with low values of the Sauter Mean Radii are airborne for long time periods so that they are able to deposit on the facades of the iconic Meenakshi Madurai temple towers. The paper does not touch upon the theme of façade restoration. Nor does it discuss or quantify any aesthetic thresholds and the blackening of facades^40^. Madurai is an important temple city, and the Meenakshi Temple has been in continuous worship for over a thousand years. Local residents as well as tourists will need to be involved in any major decision-making operations and aesthetic thresholds can be decided factoring in public perception of the acuteness of the problem so that the temple towers do not become unacceptably discoloured. The paper does not discuss the theme of restoration of the painted facades that can be undertaken in a cost-effective way.

The paper proposes a novel protocol where the expected particle size distributions of the emitted sot and black carbon from operating diesel vehicles can be easily forecast. In fact, AI mediated tools can be developed so that the pollution thresholds vis-à-vis the particle size distributions are alerted zone wise through mobile apps. In the present study the model boundary enclosed a highly congested city with varying urban density. Ideally, Geospatial modelling zooming in on specific problem areas should be used along with WRF model for a fuller characterisation. In-situ sampling is beginning to emerge from the Pollution Control Boards of several states in India including Tamil Nadu. Such data must be used as and when they are available. These will form part of a future study.

The arrangement of sculptures on the towers is non-homogeneous and the towers taper upwards calling for a fuller analysis involving aerodynamic processes that might operate. These concerns are only beginning to emerge. Normally, the size of particulate pollutants is very small, they are easily deposited onto the horizontal and sloping surface of stone structures under the action of aerodynamics processes^41^. These considerations will also form part of our future studies.

## Wider implications

The results shown in this paper, for the first time, gives an accurate projection over several decades (2020–2050) of five useful parameters (Surface Temperature, Leaf Area Index, Cloud Cover, Soil Moisture and Precipitation) useful for environmental impact analysis and for better planning and regulation of urban environments around heritage sites. It was shown that the decrease of the LAI values during 1940–2000 could be attributed to the build-up of urban density over the region. By 2020 when this reached a tipping point, without any scope of further urbanisation, the rate of decrease of the LAI from 2020 right up to 2060 slowed down. This marked increase/decrease trend was in sync with the variability of Temperature, Soil Moisture, Precipitation and Cloud Cover. It was also shown that the prevailing Soot and Black Carbon (BC) concentrations had Sauter Mean Radii smaller than 1 micron—at this size range they have a propensity to have atmospheric residence times over a week and contribute to residual pollution^19,20^. These particles can easily settle on to the temple facades. With a clear identification of the SMR and range of sizes involved, the most obvious and compelling remediation strategy in current times (until such time when EVs have not replaced diesel vehicles) must involve the use of special sprinklers. Here, there is a caveat—without a judicious choice, often sprinklers have failed to wash out smog—the collector droplets emanating from sprinkler heads were too large and settled fast with large Stokesian settling velocities. In contrast, when an ultrafine mist was used, the collection probabilities of these ‘collector’ droplets were very low because the Soot and BC particles with small SMR could not align within the aerodynamic capture distances. It is suggested that sprinklers are used which release a droplet mist with radii about 5–10 times the SMR encountered over the region as shown in Table 2.

To put forth a convincing argument in favour of diesel vehicle replacement in the coming decade, one must quantitatively account for the amounts of soot and black carbon deposited on to a prominent temple gopuram. Thereafter, it is straightforward to estimate costs of restoration using a Transformer Model.

**Fig. 13:**
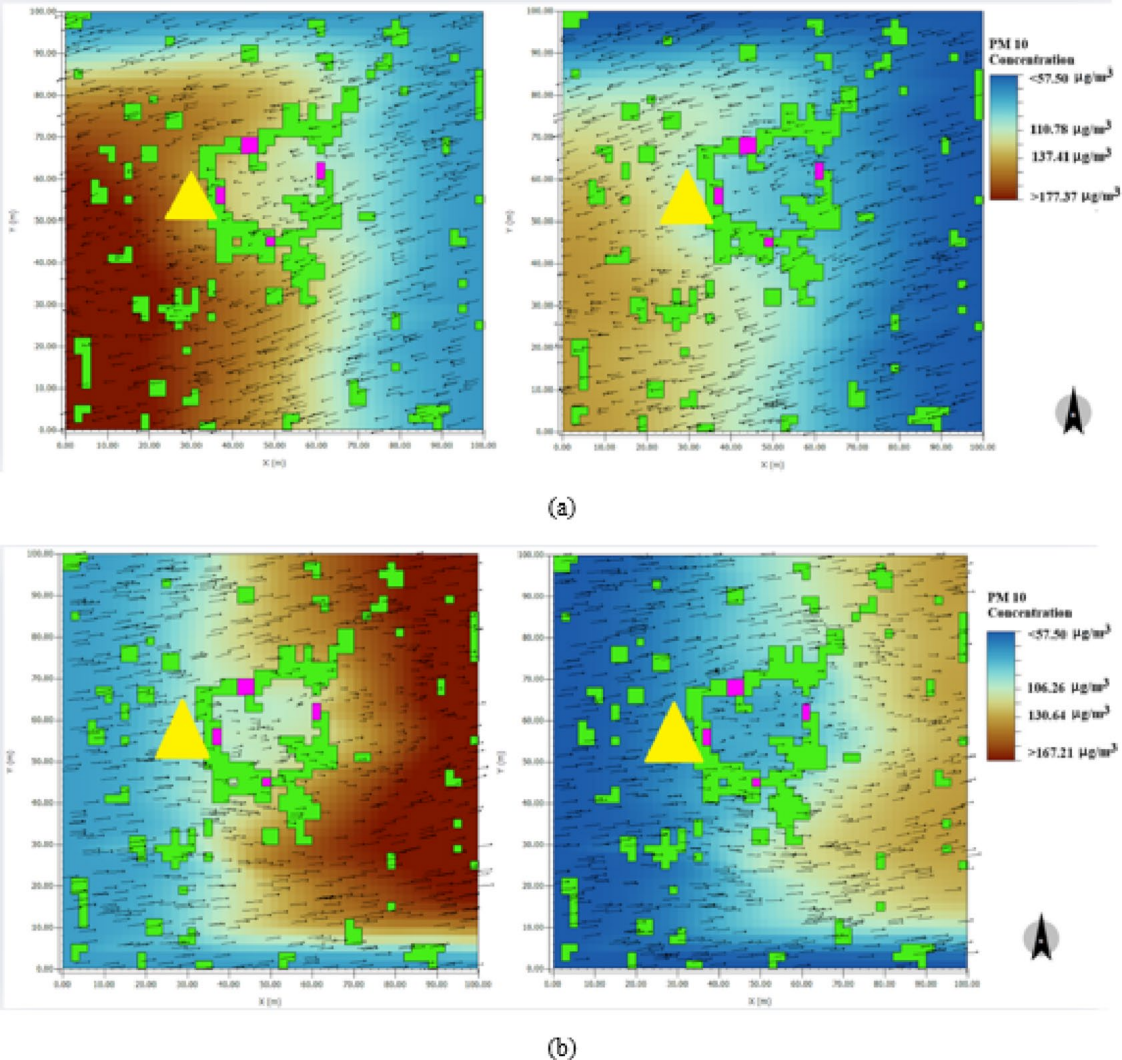
PM 10 deposition curve (**a**) 15th February 2021, (**b**) 15th July 2021 with (Right Panel) and without EV (Left Panel). The yellow triangle represents the Tower gate.

It was possible to have a handle on the PM10 loading over a year, on the western façade, which is the most vulnerable. We describe two cases, 1—Business as usual case with all peripheral roads plying diesel vehicles and case 2 with a phasing out of diesel vehicles and the use of Electric Vehicles (EVs). The above was achieved with the—ENVI-met run for the months of February and July 2021 to assess the effect of deposition during months when the wind directions reversed. Feb is a relatively cool month with a build-up of pollution whilst July being a SW monsoon period month there are wash out effects. In the Fig. 13, the left panels pertain to the business-as-usual case, and the right panels correspond to the case when all diesel vehicles are replaced with Electric Vehicles. The ambient PM10 concentrations in the former case are much higher than what obtains for the situation when EV’s are used. With such high airborne values, apart from the adverse health effects, one must note that the Meenakshi Temple area is a protected zone. With an almost saturated built-up area in the surroundings, and with progressively worsening LULC changes, one must look for immediate solutions. Earlier, we have discussed the usefulness of using optimised sprinklers using the Sauter Mean Radii values shown in Table 2. Whilst, this is a relatively straightforward solution, a long-term solution must involve a phased-out replacement of diesel vehicles with Electric Vehicles. With the above in perspective, we show Envi-met results for the latter case shown on the right panels of Fig. 13. One observes a 42% reduction in the PM10 concentrations.

**Fig. 14:**
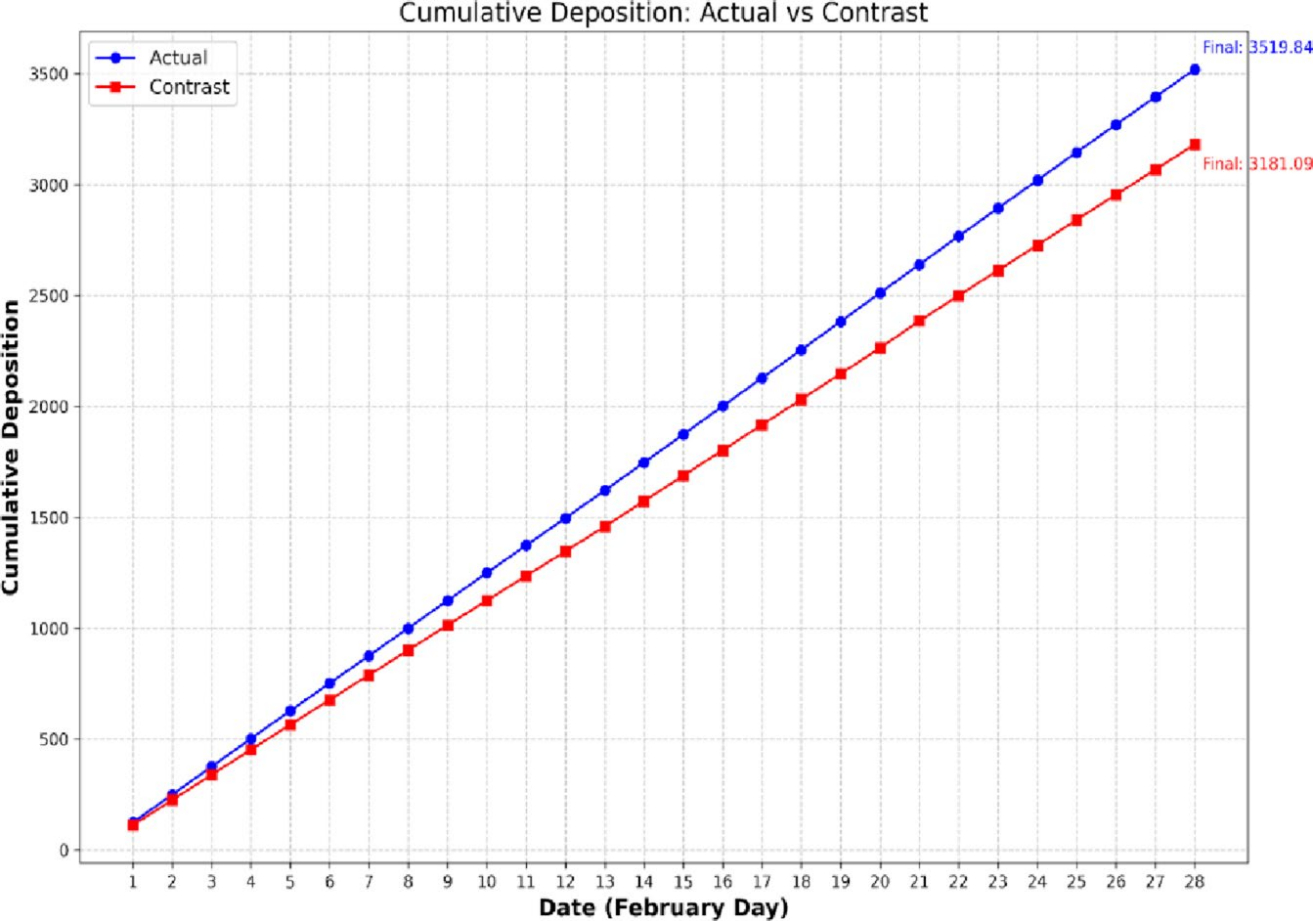
PM 10 Deposition on the West Gopuram of the Meenakshi Temple during the month of February when deposition was high. A transition to EVs shows a clear 10% decrease just on the west gopuram over a month.

Figure 13b shows a 34% decrease in the cumulative PM10 deposition for the month of July which impacts the iconic western gopuram of the Meenakshi Temple complex when diesel vehicles are substituted by EVs. The extent of cumulative deposits are shown in Fig. 14.

From Fig. 14, upon extrapolating cumulatively over a period of 5 years, one easily finds that the deposits will exponentially increase. From estimates of the surface area of each gopuram, the Intricacy Factor (effectively a ratio of actual exposed surface area to projected flat surface area, with flat stucco surfaces serving as the baseline), the cost per square foot of cleaning twice a year, adds up to several hundred thousand in USD. This will be explored fully in a subsequent paper.

This case study for a 2000-year-old continuously worshiped and well-loved temple may well pave the way for several other similar studies over other South Asian cities which house a plethora of heritage sites in the close vicinity of urban sprawl and badly managed vehicular traffic.

## Supplementary Information

Below is the link to the electronic supplementary material.

Supplementary Material 1

## Data Availability

Data used in this study are sourced from: ECMWF Reanalysis v5 (ERA5) DatasetWeather Research and Forecast Model Output dataMESACLIP: A 10-Member Ensemble of CESM HR RCP 8.5 Simulations Sourced from MESACLIP DatasetInput data for Transformer model were sourced from cds.climate.copernicus.euland.copernicus.euopen-meteo.com/en/docs/historical-weather-api.
