# Supplementary material for: Novel R-CNN and transformer models for pollution impacts and land cover changes around iconic heritage sites in developing countries: a case study
Source: Sci Rep. 2025 Dec 3;15:43134. doi: 10.1038/s41598-025-27155-x (PMC12678472; doi:10.1038/s41598-025-27155-x)
Supplement: Supplementary file 1 — Supplementary Material 1 [file 41598_2025_27155_MOESM1_ESM.docx]

**Supplementary File A (MATLAB Code)**

% Example: Transformer-based prediction (MATLAB)

clc; clear; close all;

% Load Historical Data

data = readtable('Historical_Data_Sourced.xlsx');

%data = readtable('Updated_Dataset_with_50__EV_Adoption.csv');

years = data.Year;

X_all = years(:); % Ensure column vector

Y_all = data{:, 2:end};

%% STEP 1: Split into Training and Validation Sets

splitRatio = 0.7;

N = length(X_all);

idx = randperm(N);

trainIdx = idx(1:round(splitRatio*N));

valIdx = idx(round(splitRatio*N)+1:end);

XTrain = X_all(trainIdx);

YTrain = Y_all(trainIdx, :);

XVal = X_all(valIdx);

YVal = Y_all(valIdx, :);

%% STEP 2: Normalize Based on Training Set Only

[XTrainNorm, minX, maxX] = normalizeData(XTrain);

[YTrainNorm, minY, maxY] = normalizeData(YTrain);

XValNorm = normalizeData(XVal, minX, maxX);

YValNorm = normalizeData(YVal, minY, maxY);

%% Define Transformer Parameters

numFeatures = size(YTrain, 2);

numHeads = 4;

numLayers = 5;

dModel = 64;

dff = 128;

dropoutRate = 0.1;

% Define Transformer Layers

layers = [

sequenceInputLayer(1, 'Name', 'input')

fullyConnectedLayer(dModel, 'Name', 'embedding')

addTransformerBlock(dModel, numHeads, dff, dropoutRate, 'transformer1')

addTransformerBlock(dModel, numHeads, dff, dropoutRate, 'transformer2')

fullyConnectedLayer(numFeatures, 'Name', 'output_fc')

regressionLayer('Name', 'output')];

%% Define Training Options (With Validation)

options = trainingOptions('adam', ...

'MaxEpochs', 500, ...

'MiniBatchSize', 3, ...

'InitialLearnRate', 0.001, ...

'Shuffle', 'every-epoch', ...

'Plots', 'training-progress', ...

'Verbose', false, ...

'ValidationData', {XValNorm', YValNorm'}, ...

'ValidationFrequency', 10, ...

'ValidationPatience', 30);

% Train Network

net = trainNetwork(XTrainNorm', YTrainNorm', layers, options);

%% Predict Future Values

future_years = (2025:1:2050)';

future_years_norm = normalizeData(future_years, minX, maxX);

predicted_values_norm = predict(net, future_years_norm');

predicted_values = denormalizeData(predicted_values_norm', minY, maxY);

% Create Prediction Table

predicted_table = array2table([future_years, predicted_values], ...

'VariableNames', {'Year', 'LAI', 'CloudCover', 'Precipitation', 'Temperature', 'SoilMoisture'});

writetable(predicted_table, 'predicted_environmental_data_transformer.csv');

disp(predicted_table);

%% Plot Predictions vs Training Data

params = {'LAI', 'CloudCover', 'Precipitation', 'Temperature', 'SoilMoisture'};

colors = ['r', 'g', 'b', 'm', 'c'];

figure;

for i = 1:numFeatures

subplot(3, 2, i);

plot(X_all, Y_all(:, i), 'o', 'MarkerFaceColor', colors(i)); hold on;

plot(future_years, predicted_values(:, i), '-x', 'Color', colors(i), 'LineWidth', 1.5);

title(params{i}); xlabel('Year'); ylabel(params{i});

legend('Actual', 'Predicted'); grid on;

end

%% Transformer Block Function

function layer = addTransformerBlock(dModel, numHeads, dff, dropoutRate, name)

layer = [

fullyConnectedLayer(dModel, 'Name', [name '_query'])

fullyConnectedLayer(dModel, 'Name', [name '_key'])

fullyConnectedLayer(dModel, 'Name', [name '_value'])

fullyConnectedLayer(dModel, 'Name', [name '_attention'])

layerNormalizationLayer('Name', [name '_ln1'])

dropoutLayer(dropoutRate, 'Name', [name '_dropout1'])

fullyConnectedLayer(dff, 'Name', [name '_fc1'])

reluLayer('Name', [name '_relu'])

fullyConnectedLayer(dModel, 'Name', [name '_fc2'])

layerNormalizationLayer('Name', [name '_ln2'])

dropoutLayer(dropoutRate, 'Name', [name '_dropout2'])];

end

%% Normalization Helper

function [normalizedData, minVal, maxVal] = normalizeData(data, minVal, maxVal)

if nargin < 2

minVal = min(data, [], 1);

maxVal = max(data, [], 1);

end

normalizedData = (data - minVal) ./ (maxVal - minVal + eps);

end

%% Denormalization Helper

function denormalizedData = denormalizeData(normalizedData, minVal, maxVal)

denormalizedData = normalizedData .* (maxVal - minVal + eps) + minVal;

end

**Supplementary File B (Python algorithm with sample SEM images)**

**Input:**

- Reference images R={R1​,R2​,…,Rk​}
- Test image T
- Parameter set Θ

**Output:**

- Particle set P
- Size distribution summary D

1. **Initialize Descriptor Bank**
   B←∅
2. **Reference Descriptor Extraction**
   For each Ri​∈R:
     a. Fi​←Preprocess(Ri​,Θp​)
     b. Di​←ExtractDescriptors(Fi​,Θd​)
     c. B←B∪Di​
3. **Reference Consolidation**
   B∗←ConsolidateReference(B)
4. **Test Image Preparation**
   FT​←Preprocess(T,Θp​)
   FT​←ApplyMask(FT​,Θm​)
5. **Candidate Generation**
   C←FindCandidates(FT​)
6. **Reference-Guided Filtering**
   P←∅
   For each c∈C:
     a. dc​←ComputeDescriptor(c)
     b. If IsMatch(dc​,B∗,Θf​)=True:
       Add c to P
7. **Physical Calibration**
   For each p∈P:
     Compute rp​=EquivRadius(p)⋅Θc​
8. **Distribution Estimation**
   D←SummarizeDistribution({rp​},Θs​)
9. **Return Results**
   Return (P,D)

Example of a SEM image from Experimental Analysis:

**
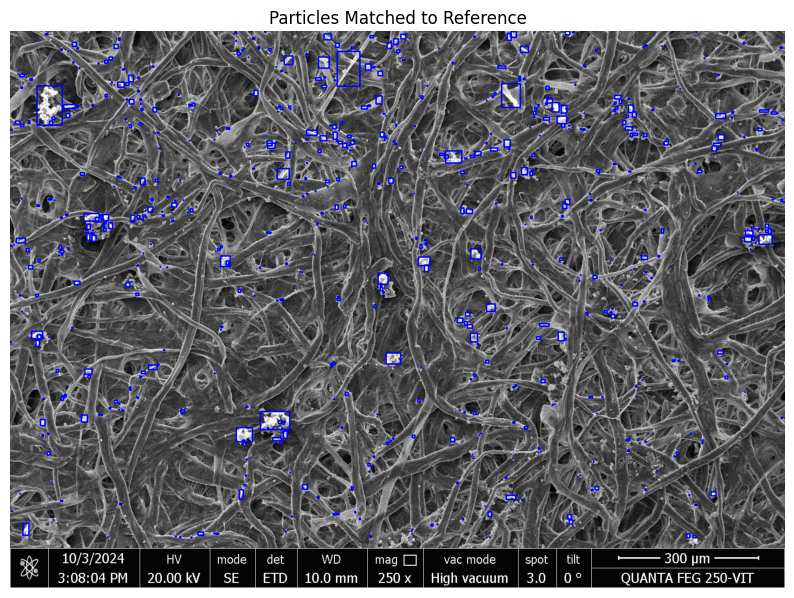
**
